# 

**Published:** 1803-09-01

**Authors:** 


### TO CORRESPONDENTS.

Some of our Readers have signified that the concluding paragraph of the Review of the *Report of the Vaccine Pock Institution* near Golden Square, (page 190 of our last number) may admit of an interpretation injurious to both the parties alluded to; we have therefore given the whole passage from the Report, that our readers may put their own construction upon it.

"The Public have been told, even in terms of invective, and reports have been industriously propagated, that the Founders have been guilty of a flagrant act of injustice in excluding Dr. Jenner from this Institution. If our adversaries had but been thoughtful, candid, and judicious enough to have ascertained the truth of the supposed fact, to which their censures were imputed, they would have spared themselves the trouble of going further, granting (which we are not required in propriety to do) that it was an act of injustice to do what was asserted The truth is, that Dr. Jenner was invited by one of your Reporters, Dr. Pearson, in a letter addressed to him at Be kely, his place of residence, December 10, 1799, to belong to the Institution, under the most honourable title then deemed consistent, that of consulting or corresponding physician. This proposal was declined, without intimating that any other situation would be more agreeable. Subsequently, in order to shew due respect and deference to some friends of the Institution, Mr. Brande was deputed by the Vaccine Medical Committee to wait upon Dr. Jenner, then in town, and assure him of the willingness of each and all the medical department to make any arrangement agreeable to induce him to be attached to the Institution; the Committee even went so far as to authorise Mr. Brande to say, Dr. Jenner might make what alterations he pleased in the plan of the Institution, and that if any of the officers were not agreeable to him, there was not one who was not willing to resign. After repeated applications and much delay in the spring 1800, all the proposals were declined, it being at last said, it was thought best, that there should be no Institution."

We have received from Mr. Moor, surgeon dentist, some Strictures on Mr. Fox's work on the Teeth. The object of the Writer is to controvert Mr. F's opinion respecting the vascularity of the teeth; but on comparing some of the quotations with Mr. F's book, we did not think them sufficiently correct for publication, and request Mr. M. to send for his paper from the publisher's, for revision.

The Editors have frequently informed their Correspondents, that anonymous answers to, or criticisms on, papers signed by the Authors, cannot be admitted, unless the Writer's address accompanies them in a postscript, or some other manner. It should hardly seem necessary to observe, that medical facts without the name of the Practitioner who observed them, are no facts whatever to the Public. They regret to see so many of their Correspondents disregard these often repeated hints; every month brings them many valuable Observations and Cases, which cannot be published for want of proper Signatures.

